# Supplementary material for: Explaining the unique nature of individual gait patterns with deep learning
Source: Sci Rep. 2019 Feb 20;9:2391. doi: 10.1038/s41598-019-38748-8 (PMC6382912; doi:10.1038/s41598-019-38748-8)
Supplement: Supplementary file 1 — Supplement [file 41598_2019_38748_MOESM1_ESM.pdf]

Explaining the unique nature of individual gait patterns with deep learning
–
SUPPLEMENTARY MATERIAL

Fabian Horst[1,+], Sebastian Lapuschkin[2,+], Wojciech Samek[2,*], Klaus-Robert Müller[3,4,5,*], and Wolfgang I. Schöllhorn[1,*]

[1]Department of Training and Movement Science, Institute of Sport Science, Johannes Gutenberg-University Mainz, Mainz, Rhineland-Palatinate, Germany
[2]Department of Video Coding & Analytics, Fraunhofer Heinrich Hertz Institute, Berlin, Germany
[3]Department of Electrical Engineering & Computer Science, Technical University Berlin, Berlin, Germany
[4]Department of Brain and Cognitive Engineering, Korea University, Seoul, Korea
[5]Max Planck Institute for Informatics, Saarbrücken, Saarland, Germany
[*]wojciech.samek@hhi.fraunhofer.de (WS)
[*]klaus-robert.mueller@tu-berlin.de (KRM)
[*]wolfgang.schoellhorn@uni-mainz.de (WIS)
[+]these authors contributed equally to this work

ABSTRACT

Machine learning (ML) techniques such as (deep) artificial neural networks (DNN) are solving very successfully a plethora of tasks and provide new predictive models for complex physical, chemical, biological and social systems. However, in most cases this comes with the disadvantage of acting as a black box, rarely providing information about what made them arrive at a particular prediction. This black box aspect of ML techniques can be problematic especially in medical diagnoses, so far hampering a clinical acceptance. The present paper studies the uniqueness of individual gait patterns in clinical biomechanics using DNNs.
By attributing portions of the model predictions back to the input variables (ground reaction forces and full-body joint angles), the Layer-Wise Relevance Propagation (LRP) technique reliably demonstrates which variables at what time windows of the gait cycle are most relevant for the characterisation of gait patterns from a certain individual. By measuring the time-resolved contribution of each input variable to the prediction of ML techniques such as DNNs, our method describes the first general framework that enables to understand and interpret non-linear ML methods in (biomechanical) gait analysis and thereby supplies a powerful tool for analysis, diagnosis and treatment of human gait.

This document contains 3 supplementary tables with results in Tables ST1-ST3, 6 supplementary tables describing neural network architectures in Tables ST4-ST9 and 4 supplementary subject specific analyses in Figures SF1-SF4.

1 Supplementary Results

Model	Ground Reaction Forces [%]	Joint Angles Full-Body [%]	Joint Angles Full-Body (flex.-ext.) [%]	Joint Angles Lower-Body [%]	Joint Angles Lower-Body (flex.-ext.) [%]
Linear (SGD)	95.4 (1.7)	100.0 (0.0)	96.3 (1.8)	100.0 (0.0)	91.5 (2.2)
Linear (SVM)	100.0 (0.0)	100.0 (0.0)	99.7 (0.4)	100.0 (0.0)	99.8 (0.6)
MLP (2, 64)	80.7 (3.6)	100.0 (0.0)	75.7 (4.7)	99.9 (0.3)	55.6 (6.5)
MLP (2, 128)	89.1 (2.9)	100.0 (0.0)	85.7 (4.7)	99.9 (0.3)	69.9 (5.7)
MLP (2, 256)	94.4 (2.3)	100.0 (0.0)	92.1 (3.2)	100.0 (0.0)	78.6 (4.2)
MLP (2, 512)	96.6 (0.8)	100.0 (0.0)	95.7 (1.2)	100.0 (0.0)	87.1 (2.6)
MLP (2, 1024)	98.6 (1.1)	100.0 (0.0)	97.0 (2.0)	100.0 (0.0)	91.9 (3.4)
MLP (3, 64)	88.3 (3.7)	99.9 (0.3)	84.3 (3.3)	99.9 (0.3)	68.5 (8.3)
MLP (3, 128)	92.5 (1.9)	100.0 (0.0)	90.9 (2.0)	100.0 (0.0)	84.2 (4.7)
MLP (3, 256)	96.6 (0.8)	100.0 (0.0)	95.6 (2.6)	100.0 (0.0)	89.4 (4.3)
MLP (3, 512)	98.2 (0.9)	100.0 (0.0)	97.0 (1.0)	100.0 (0.0)	94.0 (2.4)
MLP (3, 1024)	98.8 (1.3)	100.0 (0.0)	97.8 (1.0)	100.0 (0.0)	96.5 (1.2)
CNN–A	99.1 (0.8)	100.0 (0.0)	95.6 (1.7)	99.9 (0.3)	92.0 (3.9)
CNN–A3	99.6 (0.6)	99.8 (0.4)	95.0 (1.7)	99.9 (0.3)	94.2 (1.6)
CNN–A6	99.0 (0.8)	99.9 (0.3)	96.5 (1.3)	99.8 (0.4)	97.7 (1.4)
CNN–C3	99.2 (0.6)	100.0 (0.0)	97.7 (1.5)	100.0 (0.0)	97.0 (1.3)
CNN–C3–3	–	100.0 (0.0)	–	99.9 (0.3)	–
CNN–C6	99.2 (0.6)	99.9 (0.3)	97.2 (1.6)	100.0 (0.0)	95.6 (1.8)

Supplemental Material, Table ST1. The classification rates of the subject-classification of different models using artificial neural networks (n=57), reported in pairs of *mean* (*standard deviation*) in percent.

Model / Noise	Gaussian Random $\sigma = 0.5$	Gaussian Random $\sigma = 1$	Gaussian Random $\sigma = 2$	Salt^{-} Random	Pepper Random	Salt^{+} Random	Shot Random
Linear (SGD)	40.5 (6.0)	45.6 (3.0)	47.6 (1.8)	46.1 (2.7)	37.1 (7.0)	46.4 (3.1)	46.5 (2.9)
Linear (SVM)	7.4 (4.6)	26.2 (5.6)	39.9 (3.7)	34.0 (6.9)	5.0 (3.2)	35.9 (7.2)	35.9 (7.5)
MLP (2, 64)	19.2 (15.9)	29.3 (13.6)	38.8 (9.3)	43.1 (7.3)	16.6 (15.8)	42.9 (7.6)	42.8 (7.7)
MLP (2, 128)	14.4 (14.2)	25.9 (13.1)	36.8 (9.4)	42.0 (6.9)	11.7 (13.6)	41.8 (7.6)	41.7 (7.7)
MLP (2, 256)	10.3 (12)	21.8 (12.4)	34.9 (9.6)	40.8 (6.7)	8.1 (11.1)	40.7 (7.2)	40.5 (7.2)
MLP (2, 512)	7.6 (9.8)	19.0 (11.5)	32.5 (9.6)	39.6 (6.6)	5.7 (9.0)	39.4 (7.3)	39.5 (7.2)
MLP (2, 1024)	5.9 (8.1)	17.0 (11.1)	31.9 (9.2)	38.8 (6.5)	4.4 (7.0)	38.4 (7.2)	38.6 (7.2)
MLP (3, 64)	16.2 (14.0)	27.6 (12.6)	37.8 (9.0)	43.5 (7.1)	14.4 (13.6)	43.3 (7.4)	43.3 (7.4)
MLP (3, 128)	12.5 (12.3)	24.3 (11.9)	35.8 (8.9)	42.7 (6.9)	10.3 (11.9)	42.4 (7.4)	42.5 (7.4)
MLP (3, 256)	8.9 (9.7)	20.8 (10.9)	34.9 (8.4)	41.7 (6.8)	7.7 (9.5)	41.2 (7.4)	41.1 (7.5)
MLP (3, 512)	6.9 (8.4)	18.7 (10.4)	33.4 (8.2)	40.8 (6.8)	5.5 (7.6)	40.4 (7.1)	40.4 (7.2)
MLP (3, 1024)	5.4 (7.0)	16.7 (9.4)	32.4 (7.8)	40.0 (6.6)	4.3 (6.1)	39.4 (7.2)	39.4 (7.2)
CNN–A	6.1 (6.8)	18.2 (9.4)	33.1 (8.0)	36.9 (6.6)	8.8 (8.5)	37.4 (7.5)	37.4 (7.6)
CNN–A3	9.4 (7.7)	23.4 (9.0)	36.6 (7.4)	38.0 (7.4)	6.6 (6.8)	39.6 (6.7)	39.6 (6.7)
CNN–A6	8.6 (7.4)	22.9 (9.3)	35.9 (7.6)	38.0 (7.0)	8.8 (8.3)	39.3 (7.0)	39.3 (6.9)
CNN–C3	12.2 (8.6)	27.5 (9.4)	38.0 (7.0)	31.0 (8.5)	11.3 (8.6)	37.9 (8.2)	38.1 (8.1)
CNN–C6	5.9 (6.5)	18.3 (9.3)	33.3 (7.8)	37.2 (6.6)	8.5 (8.2)	37.3 (7.2)	37.7 (7.2)

Supplemental Material, Table ST2. The area over perturbation curve of the subject-classification of ground reaction forces of different models using artificial neural networks for different noise perturbation runs (n=57). AOPC values are reported in pairs of *mean* (*standard deviation*). Gaussian noise types add white noise additively to the input samples. Salt^{-} and Salt^{+} noise types set input variables to -1 and 1 respectively. Pepper noise replaces input variables with 0. Shot noise replaces input variables randomly chosen from the set $\{-1, 0, 1\}$.

Model	Ground Reaction Forces	Joint Angles Full-Body	Joint Angles Full-Body (flex.-ext.)	Joint Angles Lower-Body	Joint Angles Lower-Body (flex.-ext.)
Linear (SGD)	4.31 (0.25)	4.27 (0.08)	3.93 (0.12)	4.16 (0.10)	3.86 (0.15)
Linear (SVM)	0.31 (0.08)	0.56 (0.10)	0.31 (0.07)	0.48 (0.09)	0.26 (0.05)
MLP (2, 64)	1.61 (0.56)	2.47 (0.12)	1.96 (0.29)	2.11 (0.13)	1.89 (0.38)
MLP (2, 128)	1.38 (0.46)	2.27 (0.10)	1.59 (0.25)	1.91 (0.11)	1.48 (0.32)
MLP (2, 256)	1.12 (0.34)	2.09 (0.09)	1.36 (0.21)	1.74 (0.11)	1.21 (0.27)
MLP (2, 512)	0.95 (0.28)	1.93 (0.08)	1.10 (0.15)	1.60 (0.10)	0.96 (0.21)
MLP (2, 1024)	0.83 (0.21)	1.80 (0.08)	0.94 (0.12)	1.48 (0.09)	0.83 (0.18)
MLP (3, 64)	1.31 (0.34)	2.73 (0.15)	1.58 (0.17)	2.37 (0.16)	1.41 (0.23)
MLP (3, 128)	1.00 (0.23)	2.44 (0.14)	1.28 (0.13)	2.05 (0.14)	1.02 (0.17)
MLP (3, 256)	0.84 (0.18)	2.21 (0.13)	1.05 (0.10)	1.86 (0.13)	0.85 (0.12)
MLP (3, 512)	0.72 (0.15)	2.02 (0.11)	0.90 (0.08)	1.66 (0.11)	0.70 (0.10)
MLP (3, 1024)	0.63 (0.12)	1.85 (0.10)	0.77 (0.08)	1.50 (0.10)	0.61 (0.09)
CNN–A	0.30 (0.08)	0.56 (0.09)	0.35 (0.06)	0.49 (0.08)	0.32 (0.05)
CNN–A3	0.37 (0.10)	0.64 (0.11)	0.37 (0.08)	0.55 (0.09)	0.35 (0.06)
CNN–A6	0.32 (0.09)	0.64 (0.12)	0.38 (0.07)	0.58 (0.11)	0.27 (0.05)
CNN–C3	0.35 (0.08)	0.50 (0.08)	0.43 (0.08)	0.48 (0.08)	0.44 (0.08)
CNN–C3–3	–	2.74 (0.10)	–	2.32 (0.13)	–
CNN–C6	0.60 (0.12)	0.59 (0.08)	0.45 (0.08)	0.62 (0.11)	0.27 (0.05)

Supplemental Material, Table ST3. The coefficient of variation of the subject-classification of different models using artificial neural networks (n=57). Values are reported in pairs of *mean* (*standard deviation*).

2 Neural Network Architectures

Complete description of all evaluated artificial neural network architectures. The complete set of features and their specific input dimensionalities $D = C \times T$ (T = time points, C = number of channels) for which results are reported in this study are:

- Ground Reaction Forces (GRF): $D = 6 \times 101$
- Full-Body Joint Angles (FBJA): $D = 33 \times 101$
- Full-Body Joint Angles (flexion-extension) (FBJAX): $D = 10 \times 101$
- Lower-Body Joint Angles (LBJA): $D = 18 \times 101$
- Lower-Body Joint Angles (flexion-extension) (LBJAX): $D = 6 \times 101$

All multi-layer perceptron models (MLP $(\cdots)$) and Linear models are – except for the number of input and hidden neurons – uniform in structure for all evaluated feature sets. Those models also receive the input as flattened arrays (vectorization by row concatenation) shaped $T \cdot C$. with H hidden units per layer, of which the number is uniform across all layers of the model. The variable L describes the number of output labels. The MLP and Linear architectures are summarized in Table ST4.

Model	Layer 1	Layer 2	Layer 3	Layer 4	Layer 5	Layer 6
Linear (SGD)	Dense (D,L)					
MLP (2, H)	Dense (D,H)	ReLU	Dense (H,L)	SoftMax		
MLP (3, H)	Dense (D,H)	ReLU	Dense (H,H)	ReLU	Dense (H,L)	SoftMax

Supplemental Material, Table ST4. Model architectures for the linear (Linear (SGD)) and fully-connected (MLP $(\cdots)$) artificial neural networks.)

Where Dense (N,M) describes a linear / fully-connected / dense layer with N input neurons and M output neurons.

Due to construction, the output shape of a convolutional layer is determined via an interplay of layer parameters (filter size and stride parameters) and the input shapes. Since above features are characterized in a varying number of data channels, the convolutional neural network (CNN) architectures have been adapted accordingly. The following tables (Table ST5 - Table ST9) will describe the architectures of the evaluated models in details, where convolutional layers will be described as Conv(Fc, Ft|Sc, $St|H$), where Fc and Ft are the channel axis span and a time axis span of the learned convolutional filter banks respectively, Sc and St are the channel axis stride and time axis stride and H the number of learned filters (= output depth).

The CNN models can be categorized in the following groups:

- CNN-A: Models which use one layer of convolutions and have square input filter sizes configured to read all channels at once and use the same scope to read from the time axis, i.e. $Fc = Ft = C$.
- CNN-A3: These models read all input channels at once ($Fc = C$), but the scope for reading the time axis is limited to $Ft = 6$. Two layers of convolutions.
- CNN-A6: Same as CNN-A3, with the difference of $Ft = 3$ for the input layer.
- CNN-C3: Uses convolution filters shaped (Fc, Ft) = (3,3)
- CNN-C3-3: Uses convolution filters shaped (Fc, Ft) = (3,3) and a stride pattern of (Sc, St) = (3,3), i.e. there is no overlap between locations where convolutional filters are applied. This model receives inputs padded with one additional columns of zeros, to extend the time axis to 102 entries.
- CNN-C6: Uses convolution filters shaped (Fc, Ft) = (6,6)

Model	Layer 1	Layer 2	Layer 3	Layer 4	Layer 5	Layer 6	Layer 7	Layer 8	Layer 9
CNN–A	Conv (6,6|1,1|32)	ReLU	Flatten	Dense (3072,L)	SoftMax				
CNN–A3	Conv (6,3|1,1|32)	ReLU	Conv (1,3|1,1|32)	ReLU	Flatten	Dense (3104,L)	SoftMax		
CNN–A6	Conv (6,6|1,1|32)	ReLU	Conv (1,6|1,1|32)	ReLU	Flatten	Dense (2912,L)	SoftMax		
CNN–C3	Conv (3,3|1,1|32)	ReLU	Conv (3,3|1,1|32)	ReLU	Conv (2,2|1,1|32)	ReLU	Flatten	Dense (3072,L)	SoftMax
CNN–C3–3	–	–	–	–	–	–	–	–	–
CNN–C6	Conv (6,6|1,1|32)	ReLU	Flatten	Dense (3072,L)	SoftMax				

Supplemental Material, Table ST5. Model architectures for the convolutional neural network architectures for ground reaction force variables (GRF).

Model	Layer 1	Layer 2	Layer 3	Layer 4	Layer 5	Layer 6	Layer 7	Layer 8	Layer 9
CNN–A	Conv (33,33|1,1|64)	ReLU	Flatten	Dense (4416,L)	SoftMax				
CNN–A3	Conv (33,3|1,1|64)	ReLU	Conv (1,3|1,1|32)	ReLU	Flatten	Dense (3104,L)	SoftMax		
CNN–A6	Conv (33,6|1,1|32)	ReLU	Conv (1,6|1,1|32)	ReLU	Flatten	Dense (2912,L)	SoftMax		
CNN–C3	Conv (3,3|1,1|32)	ReLU	Conv (3,3|1,1|32)	ReLU	Conv (3,3|1,1|16)	ReLU	Flatten	Dense (41040,L)	SoftMax
CNN–C3–3	Conv (3,3|3,3|64)	ReLU	Conv (3,3|1,1|64)	ReLU	Conv (3,3|1,1|32)	ReLU	Flatten	Dense (6720,L)	SoftMax
CNN–C6	Conv (6,6|1,1|32)	ReLU	Conv (6,6|1,1|32)	ReLU	Conv (6,6|1,1|16)	ReLU	Flatten	Dense (24768,L)	SoftMax

Supplemental Material, Table ST6. Model architectures for the convolutional neural network architectures for full-body joint angles (FBJA).

<table>
<tr><th>Model</th><th>Layer 1</th><th>Layer 2</th><th>Layer 3</th><th>Layer 4</th><th>Layer 5</th><th>Layer 6</th><th>Layer 7</th><th>Layer 8</th><th>Layer 9</th></tr>
<tr><td>CNN–A</td><td>Conv
(10,10|1,1|32)</td><td>ReLU</td><td>Flatten</td><td>Dense
(2944,L)</td><td>SoftMax</td><td></td><td></td><td></td><td></td></tr>
<tr><td>CNN–A3</td><td>Conv
(10,3|1,1|32)</td><td>ReLU</td><td>Conv
(1,3|1,1|32)</td><td>ReLU</td><td>Flatten</td><td>Dense
(3104,L)</td><td>SoftMax</td><td></td><td></td></tr>
<tr><td>CNN–A6</td><td>Conv
(10,6|1,1|32)</td><td>ReLU</td><td>Conv
(1,6|1,1|32)</td><td>ReLU</td><td>Flatten</td><td>Dense
(2912,L)</td><td>SoftMax</td><td></td><td></td></tr>
<tr><td>CNN–C3</td><td>Conv
(3,3|1,1|32)</td><td>ReLU</td><td>Conv
(3,3|1,1|32)</td><td>ReLU</td><td>Conv
(3,3|1,1|32)</td><td>ReLU</td><td>Flatten</td><td>Dense
(12160,L)</td><td>SoftMax</td></tr>
<tr><td>CNN–C3–3</td><td>–</td><td>–</td><td>–</td><td>–</td><td>–</td><td>–</td><td>–</td><td>–</td><td>–</td></tr>
<tr><td>CNN–C6</td><td>Conv
(6,6|1,1|32)</td><td>ReLU</td><td>Conv
(3,3|1,1|32)</td><td>ReLU</td><td>Conv
(3,3|1,1|32)</td><td>ReLU</td><td>Flatten</td><td>Dense
Dense (2944,L)</td><td>SoftMax</td></tr>
</table>

Supplemental Material, Table ST7. Model architectures for the convolutional neural network architectures for full-body joint angles in the sagittal plane (flexion-extension) (FBJAX).

<table>
<tr><th>Model</th><th>Layer 1</th><th>Layer 2</th><th>Layer 3</th><th>Layer 4</th><th>Layer 5</th><th>Layer 6</th><th>Layer 7</th><th>Layer 8</th><th>Layer 9</th></tr>
<tr><td>CNN–A</td><td>Conv
(18,18|1,1|64)</td><td>ReLU</td><td>Flatten</td><td>Dense
(5376,L)</td><td>SoftMax</td><td></td><td></td><td></td><td></td></tr>
<tr><td>CNN–A3</td><td>Conv
(18,3|1,1|64)</td><td>ReLU</td><td>Conv
(1,3|1,1|32)</td><td>ReLU</td><td>Flatten</td><td>Dense
(3104,L)</td><td>SoftMax</td><td></td><td></td></tr>
<tr><td>CNN–A6</td><td>Conv
(18,6|1,1|64)</td><td>ReLU</td><td>Conv
(1,6|1,1|32)</td><td>ReLU</td><td>Flatten</td><td>Dense
(2912,L)</td><td>SoftMax</td><td></td><td></td></tr>
<tr><td>CNN–C3</td><td>Conv
(3,3|1,1|32)</td><td>ReLU</td><td>Conv
(3,3|1,1|32)</td><td>ReLU</td><td>Conv
(3,3|1,1|16)</td><td>ReLU</td><td>Flatten</td><td>Dense
(18240,L)</td><td>SoftMax</td></tr>
<tr><td>CNN–C3–3</td><td>Conv
(3,3|3,3|64)</td><td>ReLU</td><td>Conv
(3,3|1,1|64)</td><td>ReLU</td><td>Conv
(3,3|1,1|32)</td><td>ReLU</td><td>Flatten</td><td>Dense
(1920,L)</td><td>SoftMax</td></tr>
<tr><td>CNN–C6</td><td>Conv
(6,6|1,1|32)</td><td>ReLU</td><td>Conv
(6,6|1,1|32)</td><td>ReLU</td><td>Conv
(6,6|1,1|16)</td><td>ReLU</td><td>Flatten</td><td>Dense
(4128,L)</td><td>SoftMax</td></tr>
</table>

Supplemental Material, Table ST8. Model architectures for the convolutional neural network architectures for lower-body joint angles (LBJA).

Model	Layer 1	Layer 2	Layer 3	Layer 4	Layer 5	Layer 6	Layer 7	Layer 8	Layer 9
CNN–A	Conv (6,6|1,1|32)	ReLU	Flatten	Dense (3072,L)	SoftMax				
CNN–A3	Conv (6,3|1,1|32)	ReLU	Conv (1,3|1,1|32)	ReLU	Flatten	Dense (3104,L)	SoftMax		
CNN–A6	Conv (6,6|1,1|32)	ReLU	Conv (1,6|1,1|32)	ReLU	Flatten	Dense (2912,L)	SoftMax		
CNN–C3	Conv (3,3|1,1|32)	ReLU	Conv (3,3|1,1|32)	ReLU	Conv (2,2|1,1|32)	ReLU	Flatten	Dense (3072,L)	SoftMax
CNN–C3–3	–	–	–	–	–	–	–	–	–
CNN–C6	Conv (6,6|1,1|32)	ReLU	Flatten	Dense (3072,L)	SoftMax				

Supplemental Material, Table ST9. Model architectures for the convolutional neural network architectures for lower-body joint angles in the sagittal plane (flexion-extension) (LBJAX).

Supplementary Subject Specific Analyses

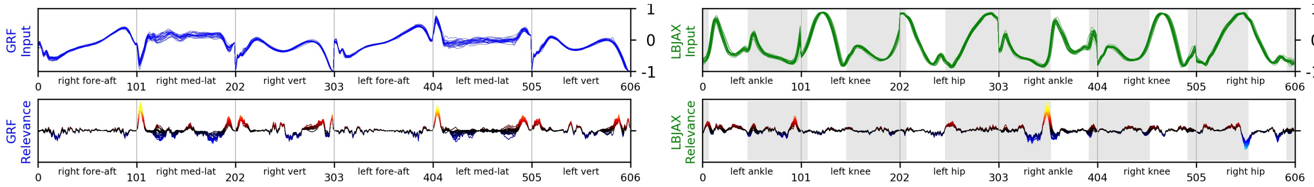

Supplemental Material, Figure SF1. Subject 21: GRF and JLBAX inputs as line plots *(top)*. The corresponding relevance maps *(bottom)* for the CNN-A, following the procedure outlined in Figure 1 in the paper. *Left:* Ground reaction force (GRF). The input relevance values point out that the model has learned to identify subject 21 by the medial-lateral shear force during the initial contact of the stance phase. The GRF input balues reveal that the medial-lateral shear forces during the initial contact of the stance phase are among the highest in the sample for both foot contacts of subject 21. *Right:* Lower-body joint angles in the sagittal plane (flexion-extension) (LBJAX). It can be observed from the input relevance values that the extension of the ankle is unique to subject 21 during the terminal stance phase of the right foot, which is identified by the model as an identifying characteristic. For this subject, the model achieves TP rates of 100% for LBJAX and 95.23% for GRF.

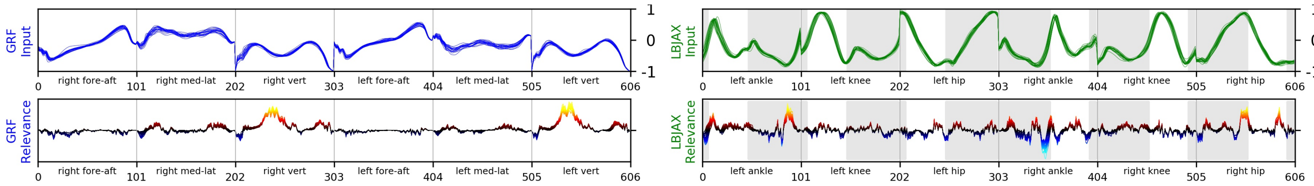

Supplemental Material, Figure SF2. Subject 50: GRF and JLBAX inputs as line plots *(top)*. The corresponding relevance maps *(bottom)* for the CNN-A, following the procedure outlined in Figure 1 in the paper. *Left:* Ground reaction force (GRF). The highest input relevance values are appearning for the vertical GRF during the mid stance phase of the right and left foot contact. This indicates that the time window of features, where the leg is located directly over the foot and the other leg is in the middle of the swing phase, is unique to subject 50. *Right:* Lower-body joint angles in the sagittal plane (flexion-extension) (LBJAX).. From the input relevance values, we can observe that the extension of the ankle during the terminal stance phase of the left leg as well as the flexion of the hip joint during the terminal stance phase and the mid swing phase of the right leg is unique to subject 50. For this subject, the model achieves TP rates of 100% for GRF but only 80% for LBJAX, for which it consistently mispredicts as subject 4. The model's uncertainty is reflected in the comparatively noisy and piece-wise negative relevance values. Relevance feedback such as for subject 50 and LBJAX data, providing detailed insight into the reasoning behind the model's uncertain prediction, yields information critical for the practical applicability of gait analysis tools based on machine learning in a clinical setting.

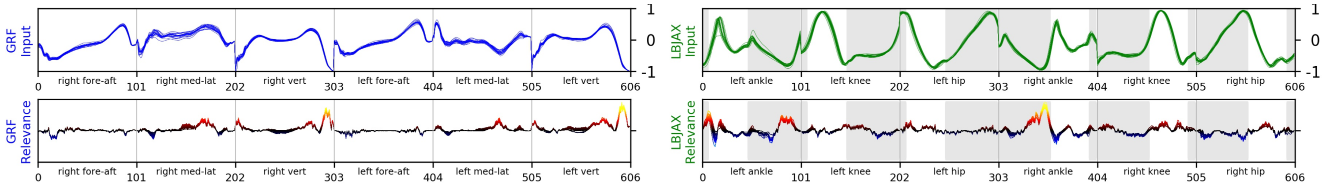

Supplemental Material, Figure SF3. Subject 54:GRF and JLBAX inputs as line plots *(top)*. The corresponding relevance maps *(bottom)* for the CNN-A, following the procedure outlined in Figure 1 in the paper. *Left:* Ground reaction force (GRF). The input relevance values point out that the model has learned to identify subject 54 based the vertical GRF during the terminal stance phase of the right and left leg. *Right:* Lower-body joint angles in the sagittal plane (flexion-extension) (LBJAX). The LBJAX supports the finding about the unique nature of the terminal stance phase to subject 54, showing the highest input relevance values for the extension of the ankle during this time window of the gait cycle. For this subject, the model achieves TP rates of 95% for LBJA and 100% for GRF.

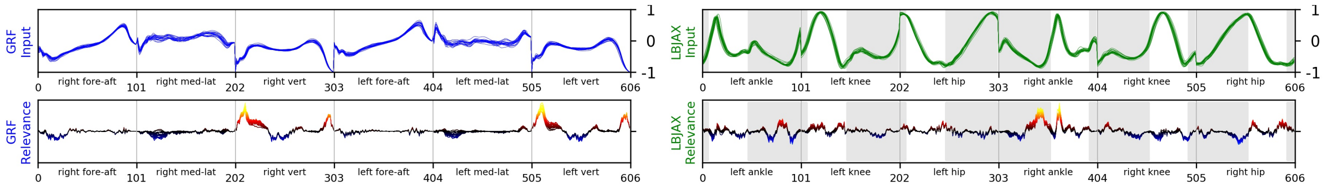

Supplemental Material, Figure SF4. Subject 56: GRF and JLBAX inputs as line plots *(top)*. The corresponding relevance maps *(bottom)* for the CNN-A, following the procedure outlined in Figure 1 in the paper. *Left:* Ground reaction force (GRF). From the input relevance values, we can observe that the vertical GRF during the initial and terminal stance phase of the right and left leg is unique to subject 56. *Right:* Lower-body joint angles in the sagittal plane (flexion-extension) (LBJAX). From the input relevance values, we can observe that the extension of the ankle during the terminal stance phase of the right is unique to subject 56. For this subject, the model achieves TP rates of 100% for both LBJAX and GRF.
